# Editorial: Development of novel engineered biomaterials with clinical translation prospects for chronic disease theranostics

**DOI:** 10.3389/fbioe.2026.2017353

**Published:** 2026-10-21

**Authors:** Hu Chen, Huirong Lin, Xiaoliu Liang, Jianzhong Zhang, Chengchao Chu

**Affiliations:** 1 Department of Hepatobiliary Surgery, First Affiliated Hospital of Xi’an Jiaotong University, Xi’an, China; 2 Brigham and Women’s Hospital, Harvard Medical School, Boston, MA, United States; 3 Guangxi Medical University Cancer Hospital, Guangxi Medical University, Nanning, China; 4 Department of Neurological Surgery, Feinberg School of Medicine, Northwestern University, Chicago, IL, United States; 5 Xiamen University Affiliated Xiamen Eye Center, Eye Institute of Xiamen University, School of Medicine, Xiamen University, Xiamen, China

**Keywords:** biomaterials, biomedical engineering, cancer, chronic diseases, chronic respiratory disease, diabetes, molecular image, theranostics

Chronic diseases such as cancer, cardiovascular disease, diabetes, and chronic respiratory disorders continue to impose an immense global health burden. Although molecular diagnostics and targeted therapies have advanced considerably, persistent limitations in precision, long-term disease management, and patient quality of life highlight the need for new translational strategies (Chen et al., 2020; Zhang et al., 2023). Engineered biomaterials have emerged as a promising bridge between materials science, biomedical engineering, and clinical medicine (Liang et al., 2024; Chen et al., 2022). By integrating diagnostic and therapeutic functions, controlling drug release, improving targeting, and reducing systemic toxicity, these materials offer new ways to address chronic disease theranostics (Cai et al., 2026; Lin et al., 2021). This Research Topic, “Development of novel engineered biomaterials with clinical translation prospects for chronic disease theranostics,” was launched to collect work that prioritizes clinical feasibility, biological safety, and reproducible fabrication.

A critical prerequisite for clinical translation is the ability to model and understand the diseased tissue microenvironment. The contribution (Preuß et al.) on decellularized lung scaffolds addresses this need in the context of interstitial lung diseases such as idiopathic pulmonary fibrosis and fibrotic hypersensitivity pneumonitis. The extracellular matrix is a key regulator of tissue homeostasis and remodeling, yet generating structurally intact scaffolds from fibrotic human lungs remains difficult. Existing protocols often remove cells incompletely or disrupt important matrix components, and most rely on agarose embedding for thin lung slices. The reported agarose-free, apoptosis-assisted approach uses camptothecin and raptinal together with a mild detergent, and introduces a gelatin-based temporary cutting support that is completely removable before recellularization. This standardized decellularized lung scaffold platform removes more than 99.8% of nuclei in both fibrotic and non-fibrotic tissue while preserving collagen and elastin, as confirmed by semi-quantitative image analysis. Importantly, the scaffolds support repopulation with primary human lung fibroblasts, with non-fibrotic scaffolds showing pronounced contraction and fibrotic scaffolds retaining their dimensions. Although fibroblast viability on the scaffolds was lower than on plastic, cytotoxicity remained acceptable. This work provides a physiologically relevant platform for studying matrix–cell interactions and for therapeutic screening in interstitial lung disease models, directly supporting the Topic’s emphasis on clinically translatable biomaterials.

If the lung scaffold study represents a foundation for disease modeling and preclinical screening, the next contribution (Wang et al.) moves toward active therapeutic intervention by modulating the chronic wound microenvironment. Diabetic wounds are characterized by persistent oxidative stress, prolonged inflammation, impaired angiogenesis, and disrupted bioelectric cues, all of which hinder re-epithelialization. The injectable Prussian Blue nanofiber–PVA hydrogel described in this collection combines electroconductivity with immunomodulatory features. Electrospun Prussian Blue nanofibers are uniformly embedded within a physically crosslinked PVA matrix, producing a flexible and adhesive composite with stable conductivity. *In vitro*, the hydrogel shows excellent cytocompatibility, reduces pro-inflammatory cytokines such as IL-6, TNF-α, and CD86, and enhances pro-regenerative markers including CD206 and CD31. In streptozotocin-induced diabetic mice, it accelerates wound closure, reduces inflammatory infiltration, and promotes collagen deposition and CD31-positive staining. The authors appropriately note that reactive oxygen species levels and endogenous wound electrical fields were not directly quantified, so mechanistic interpretations remain plausible and require further validation. Nevertheless, this multifunctional dressing illustrates how engineered biomaterials can be designed to reshape a hostile chronic disease microenvironment and improve healing outcomes.

Beyond local tissue repair, the collection also addresses systemic delivery and theranostic applications through extracellular vesicles (Zou et al.) and liver-targeted nanoparticles (Peng et al.). Extracellular vesicles have emerged as pivotal mediators of intercellular communication and promising naturally derived nanotherapeutics. They possess stable physicochemical properties, low immunogenicity, and inherent biocompatibility, and increasing attention is being directed toward clinical investigation. Several extracellular vesicle-based diagnostic and therapeutic products have begun to emerge, yet persistent technical and translational hurdles continue to impede clinical implementation. The review in this Topic critically discusses completed and emerging clinical investigations and proposes strategic directions to accelerate translation. In parallel, the review on nanoparticle technologies for liver targeting highlights the liver’s natural tropism for circulating nanoparticles and its central role in detoxification. Upon systemic administration, nanoparticles accumulate in the liver and are internalized by hepatic macrophages, sinusoidal endothelial cells, and hepatocytes. Understanding hepatic sinusoidal physiology and these cellular interactions is essential for optimizing liver-targeted delivery systems and improving accumulation at pathological sites. Together, these two contributions extend the collection from local microenvironment modulation to systemic diagnosis and treatment, and from naturally derived carriers to synthetic organ-targeted platforms. They reinforce a central message of the Topic: successful clinical translation depends not only on material design but also on a thorough understanding of biological barriers, delivery routes, and disease-specific pathophysiology.

Collectively, the contributions to this Research Topic form a logical continuum rather than a simple catalogue of independent studies. They move from disease-mimicking extracellular matrix platforms, to microenvironment-modulating hydrogels, to systemically delivered nanotherapeutics and diagnostics. Across this continuum, several shared requirements for clinical translation become clear: reproducible and standardized fabrication, preservation of biological function, favorable safety profiles, mechanistic validation, and feasibility for scale-up. The studies also remind us that translation is rarely achieved through a single material innovation; it requires iterative dialogue among materials scientists, biologists, clinicians, and regulatory experts. Future progress will depend on validating long-term safety and efficacy, refining patient-specific models, and developing manufacturing and quality-control standards that can meet clinical demands. We hope this collection will inspire further research that bridges laboratory discovery and clinical application, ultimately improving outcomes for patients living with chronic diseases. We thank all authors, reviewers, and editorial staff for their valuable contributions to this Topic.
